# Wealth inequalities in reproductive and child health preventive care in Mozambique: a decomposition analysis

**DOI:** 10.1080/16549716.2022.2040150

**Published:** 2022-03-15

**Authors:** Chanvo S. L. Daca, Barbara Schumann, Carlos Arnaldo, Miguel San Sebastian

**Affiliations:** a Department of Cooperation, Ministry of Health, Directorate of Planning and Cooperation, Maputo, Mozambique; b Department of Epidemiology and Global Health, Umeå University, Umeå, Sweden; c Universidade Eduardo Mondlane, Maputo, Mozambique

**Keywords:** Socioeconomic inequality, decomposition analysis, health preventive care, Mozambique

## Abstract

**Background:**

Assessing the gap between rich and poor is important to monitor inequalities in health. Identifying the contribution to that gap can help policymakers to develop interventions towards decreasing that difference.

**Objective:**

To quantify the wealth inequalities in health preventive measures (bed net use, vaccination, and contraceptive use) to determine the demographic and socioeconomic contribution factors to that inequality using a decomposition analysis.

**Methods:**

Data from the 2015 Immunisation, Malaria and AIDs Indicators Survey were used. The total sample included 6946 women aged 15–49 years. Outcomes were use of insecticide-treated nets (ITN), child vaccination, and modern contraception use. Wealth Index was the exposure variable and age, marital status, place of residence, region, education, occupation, and household wealth index were the explanatory variables. Wealth inequalities were assessed using concentration indexes (Cindex). Wagstaff-decomposition analysis was conducted to assess the determinants of the wealth inequality.

**Results:**

The Cindex was −0.081 for non-ITN, −0.189 for lack of vaccination coverage and −0.284 for non-contraceptive use, indicating a pro-poor inequality. The results revealed that 88.41% of wealth gap for ITN was explained by socioeconomic factors, with education and wealth playing the largest roles. Lack of full vaccination, socioeconomic factors made the largest contribution, through the wealth variable, whereas geographic factors came next. Finally, the lack of contraceptive use, socioeconomic factors were the main explanatory factors, but to a lesser degree than the other two outcomes, with wealth and education contributing most to explaining the gap.

**Conclusion:**

There was a pro-poor inequality in reproductive and child preventive measures in Mozambique. The greater part of this inequality could be attributed to wealth, education, and residence in rural areas. Resources should be channeled into poor and non-educated rural communities to tackle these persistent inequities in preventive care.

## Background

There is a large amount of literature revealing the importance of different socioeconomic factors for child and reproductive health outcomes. In these studies, wealth appears to be the most frequently named and relevant determinant for these outcomes [1–5]. Assessing the gap between rich and poor in health is important to monitor inequalities in population health. Identifying the demographic and socioeconomic factors that contribute to that gap can additionally help policymakers to develop targeted interventions towards decreasing that difference. Nevertheless, studies from sub-Saharan Africa (SSA) focusing on those factors are still not so common [6–9]. For instance, an SSA multi-country study about socioeconomic inequalities in immunisation revealed a pro-rich inequality, with maternal education being the largest contributor to the wealth inequality in under-five mortality [10]. In another study from Nigeria, being illiterate and living in the Northern region were the main contributing factors, besides poverty, to the observed wealth inequality in access to skilled birth attendance [11].

Mozambique has achieved modest progress in reproductive and child health indicators in the last three decades. For instance, the World Bank latest estimates indicate that the maternal mortality ratio was 289 per 100,000 live births in 2017 [12] compared to 510 per 100,000 live births in 2013 in SSA [13]. Although contraceptive coverage improved from 11.3 to 25.3% between 2011 and 2015 [14], it is still much lower than in many other African countries (around 59.3% in 2015) [15]. Furthermore, a recent study reported that the ITN utilization among children under five has increased from 36% to 73% in 2011 and 2018 respectively. Also, the proportion of children receiving measles and DPT-3 vaccination increased from 60 to 82% between 1997 and 2015 [16,17]. While some research on socioeconomic risk factors for reproductive and child health has been conducted in Mozambique [18–23], to our knowledge, only two studies have focused on decomposing the wealth inequalities in health outcomes, both pointed towards an inequality concentrated among the poor. The first one revealed that 60% of the wealth inequality in child malnutrition during 1996 to 2011 was explained by insufficient food consumption and protein intake; the second one examined wealth inequality in skilled birth attendance in 2017, identifying household poverty, low maternal education and living in a rural area as the main contributors to the gap [24,25]. Thus, socioeconomic and geographical factors seem to contribute to wealth inequalities in maternal and child health in Mozambique, despite generally free access to most of these preventive measures [21].

The objectives of this study were to quantify the wealth inequalities in reproductive and child health preventive measures (bed net, vaccination, and contraceptive use) in Mozambique and to determine the contribution of a broad range of demographic and socioeconomic factors to that inequality using a decomposition analysis.

## Methods

### Study setting

Mozambique, located in south-eastern Africa, has an estimated population of around 29 million inhabitants, the majority living in rural areas [26]. The proportion of people living under the poverty line has worsened from 52.8% in 2003 to 60% in 2021, whereas the unemployment rate for 2020 was 3.4% [27]. However, nearly 80% of the poor live in areas distant from basic public services, and the unemployment rate was 20.7% in 2015 [28,29].

The National Health Service is structured in four nested levels, from specialised hospitals in the four main cities to health centres and health posts at the community level. Health preventive services such as provision of insecticide-treated bed nets, child vaccinations and contraceptives are provided free of charge at level II (district hospitals) and level I (health posts and health centres) facilities [30].

### Study population and data collection

The AIDS and Malaria Indicators Survey (IMASIDA) is a countrywide household survey of men and women aged 15–59 years. The Demographic and Health Survey Program (DHS) conducted this survey in Mozambique from June to September 2015. A three-stage multistage cluster sampling design was used to provide representative national and province-level estimates, with stratification for rural and urban areas within provinces [17].

This process resulted in a selection of 7,368 households, out of which 7,169 participated in the study. From these households, 6,946 women of reproductive age (15–49 years) were interviewed (95% response rate) all of whom were included in the analytical sample for contraceptive use. In the analyses for two of three outcomes of our study, namely insecticide-treated nets and vaccination, the sample size was reduced to 4,709 and 2,694, respectively, due to the exclusion criteria implied in the definition of outcomes described below.

Detailed methodological procedures of the survey have been previously described. The data are publicly available and were downloaded with permission from the Demographic and Health Survey at www.dhsprogram.com/data/available-datasets.cfm.

### Survey instrument

The IMASIDA data were collected during face-to-face interviews using three questionnaires: the household, the women’s, and the men’s questionnaire. For the purpose of this study, only the women’s questionnaire was used. This questionnaire collected data on age, place of residence, marriage, occupation, education, wealth, vaccination of children, family bed net use, antenatal care, reproductive history, use of contraceptive methods, recent sexual activity, and fertility preferences. Portuguese was the language used in the interviews, and all the survey instruments were pre-tested in urban and rural areas.

### Outcome variables

Three different outcomes were used in this study capturing the lack of access to preventive health measures: Use of insecticide treated bed nets (ITN) for children, full child vaccination, and modern contraceptive use. These specific outcomes were selected because they are key monitoring indicators of the sustainable development goal 3 in the country.

Lack of ITN use was categorized as either ‘yes’ if at least one child under five had not slept under an ITN the day before the survey, or as ‘no’ if all children had slept under a bed net. For vaccination, we only included the youngest child of each woman, aged 12–59 months. The child was considered fully immunised if it had received all the recommended doses and vaccines according to the national immunisation schedule [16,17]: Bacille Calmette-Guérin (BCG) (birth dose), three doses of DPT, three doses of polio vaccine and one dose of measles vaccine. The child was classified as ‘not fully immunised’ if any of the recommended doses could not be verified by a card or reported by the mother.

Lack of modern contraceptive use was captured by asking the respondent if she had used any contraceptive methods at the last intercourse. If the answer was yes, then the woman was asked which methods she had used. Lack of modern contraceptive use was categorized as either ‘yes‘ if the woman had not used a modern contraceptive, or as ‘no’ if she had used a modern contraceptive, based on the WHO definition of modern contraceptive methods [31]. Modern contraceptives included female and male sterilisation, implants (Norplant), contraceptive pills, injectables (Depo-Provera), intrauterine contraceptive device and condoms. We classified as non-modern methods the following: periodic abstinence (rhythm, calendar method), withdrawal (coitus interruptus) and folk methods. If a respondent reported using both a modern and a non-modern method, this was counted as modern method use. In this study, modern contraceptives will be referred to as contraceptives here and after.

### Socioeconomic indicator

The variable used to depict socioeconomic status was the wealth index, obtained by principal component analysis. This was calculated based on the following assets in the participant’s household: television and car; dwelling characteristics such as flooring material; type of drinking water source; toilet facilities. The variable was used as continuous for calculating the concentration index [32].

### Explanatory variables

Three groups of variables were considered: sociodemographic (age, marital status), geographic (region and place of residence) and socioeconomic (education, occupation and wealth), based on the availability of data and their health relevance according to the literature [33].

Regarding the sociodemographic factors, age of the mother was categorised in three groups (15–24, 25–39, 40–49 years old) and marital status was divided into single/never in union, married (married/living with partner), and others (widowed, divorced, no longer living together).

Geographical factors included place of residence (dichotomised into rural or urban residence), and the three administrative regions: Northern, Central and Southern.

Maternal education was classified in three categories: no education, completed primary school, and completed secondary school or above. Nine categories of maternal occupation were captured in the IMASIDA survey, but due to the low sample size of some categories, four groups were created: (a) non-manual (managerial, clerical, sales, and services), (b) farmers, (c) manual (household and domestic, skilled, and unskilled manual) and (d) not working. The wealth index was categorised for the decomposition analysis into quintiles, the richest being the reference group [34].

### Statistical analysis

Descriptive statistics were calculated for all explanatory variables and the three outcomes.

The wealth inequality in the health preventive measures was quantified by the concentration index (Cindex), calculated based on the cumulative percentage of health preventive care and the population ranked from the poorest to the richest. The Cindex is defined as twice the area between the concentration curve and the line of equality (45-degree line) and its value can vary between −1 to +1. Concentration curves (CC) were created to illustrate the inequality for each outcome.

The Cindex can be computed as twice the covariance of the health variable and a person’s relative rank in terms of the socioeconomic status, divided by the variable mean according to the equation.

(1) $$Cindex=\frac{2}{\mu }cov\left(Yi,\;Ri\right),$$

where Cindex is concentration index; Yi the health preventive care utilisation measure; Ri the fractional rank of individual *i* in the distribution of wealth positions; μ is the mean of the health preventive care variable of the sample and cov denotes the covariance.

A negative value of the concentration index implies that a variable (here: the preventive health measure) is concentrated among the poor (pro-poor inequality), while the opposite is the case for a positive value (pro-rich inequality). The value of Cindex measures the severity of the wealth inequality in the outcome, the larger the absolute value of Cindex, the greater the inequality. When there is no inequality, the CI will be zero [35].

The CC plots the cumulative percentage of the health outcome (y-axis) against the cumulative percentage of the population, ranked by the wealth index (x-axis). If the health outcome takes a higher (lower) value among poorer people, the concentration curve will lie above (below) the line of equality.

### Decomposition of the concentration index

To determine the contribution of each sociodemographic, geographic, and socioeconomic determinant to the observed wealth inequality in each health preventive measure, a Wagstaff decomposition analysis of the Cindex was conducted.

The total Cindex can be decomposed into the contributions of k social determinants, in which each contribution is obtained by multiplying the sensitivity of the health outcome variable with respect to the determinant and the degree of wealth-related inequality in that factor. Equation (2) shows that the overall wealth inequality in health preventive measures has two components, a deterministic or ‘explained’ component and an ‘unexplained’ component.

(2) $$Cindex=\sum_{k}\left(\frac{\beta_{k}{\overset{ˉ}{x}}_{k}}{\mu }\right)C_{k}+\frac{GC_{\varepsilon }}{\mu }$$

In the first component, β_k_ is the coefficient from regressing the health outcome on determinant k. When the coefficients are weighted by the frequency of the determinant using the mean of the determinant k (x_k_) and the mean of the outcome (μ), the elasticity is calculated; hence, a category that has a high (low) coefficient might have a relatively low (high) elasticity if the category has a low (high) frequency. C_k_ is the concentration index for each determinant k and interpreted in the same way as the Cindex of the outcome. The elasticity indicates how much change in the health dependant variable is associated with one unit of change in the explanatory k variable. In the second component, GCε is the generalised Cindex for the error term, representing the amount of inequality not explained by the selected factors.

Since the reproductive and child health preventive measures in this study were binary, *probit* regression models were applied to analyse the effect of determinants on the probability of the outcomes [35,36]. In order to adjust our results to the IMASIDA sampling strategy, weighting procedures were also applied. All analyses were performed with Stata version 15.

### Ethical clearance

From the DHS website [http://www.measuredhs.com], IMASIDA data were obtained for this study. These data are all anonymous and publicly available and no ethical approval was required.

## Results

### Characteristics of the study population

The prevalence of the sociodemographic, geographic, and socioeconomic characteristics by the health preventive measures of the participants is presented in Table 1.

**Table 1.** Weighted prevalence of the sociodemographic, geographic and socioeconomic characteristics by health preventive care (total sample and by lack of use)

Variables	ITN use		Full vaccination		Contraceptive use	
	Total samplen (%)	Lack of usen (%)	Total samplen (%)	Lack of usen (%)	Total samplen (%)	Lack of usen (%)
Sociodemographic factors						
Age group						
15–24	2,053 (43.18)	1,022 (49.77)	1,016 (36.38)	461(45.40)	2,874 (41.57)	2,203 (76.64)
25–39	2,091 (43.98)	1,054 (50.38)	1,454 (52.06)	675 (46.42)	2,838 (41.04)	1,973 (69.54)
40–49	611 (12.84)	349 (57.31)	323 (11.57)	156 (48.16)	1,202 (17.39)	959 (79.79)
Marital Status						
Single	687 (14.44)	357 (51.94)	168 (6.03)	75 (44.85)	1,178 (17.04)	854 (72.51)
Married	3,295 (69.30)	1,617 (49.07)	2,106 (75.41)	936 (44.46)	4,565 (66.02)	3,411 (74.73)
Other	773 (16.26)	452 (58.42)	518 (18.56)	279 (53.96)	1,171 (16.94)	869 (74.27)
Geographical factors						
Residence						
Urban	1,506 (31.66)	653 (43.39)	795 (28.46)	298 (37.49)	2,437 (35.24)	1,607 (65.93)
Rural	3,249 (68.34)	1,772 (54.54)	1,998 (71.54)	993 (49.73)	4,478 (64.76)	3,529 (78.82)
Region						
Northern	1,670 (35.13)	734 (43.93)	1,044 (37.39)	501(48.01)	2,442 (35.31)	1,899 (77.82)
Central	1,829 (38.47)	1,016 (55.53)	1,069 (38.31)	569 (53.25)	2,502 (36.18)	2,053 (82.08)
Southern	1,255 (26.40)	676 (53.84)	679 (24.31)	221(32.49)	1,971 (28.51)	1,182 (59.99)
Socioeconomic factors						
Education						
Secondary	983 (20.68)	406 (41.26)	503 (18.01)	199 (39.48)	1,576 (22.79)	958 (60.78)
Primary	2,523 (53.05)	1,284 (50.91)	1,514 (54.23)	647 (42.70)	3,544 (51.25)	2,693 (75.99)
No education	1,249 (26.27)	735 (58.87)	775 (27.76)	446 (57.57)	1,795 (25.96)	1,485 (82.74)
Occupational class*						
Non manual	788 (16.58)	371(47.06)	502 (17.98)	219 (43.64)	1,248 (18.08)	804 (64.37)
Farmers	1,080 (22.74)	610 (56.47)	669 (23.98)	366 (54.81)	1,476 (21.38)	1,177 (79.71)
Manual	269 (5.68)	123 (45.69)	172 (6.18)	78 (45.19)	399 (5.78)	254 (63.62)
Not working	2,612 (55.00)	1320 (50.54)	1,447 (51.87)	627 (43.34)	3,782 (54.77)	2,897 (76.59)
Wealth quintiles						
Richest	560 (11.78)	400 (42.17)	259 (9.28)	172 (36.13)	975 (14.09)	934 (58.79)
Richer	672 (14.13)	510 (52.38)	375 (13.44)	189 (34.09)	1,063 (15.38)	984 (69.10)
Middle	896 (18.84)	523 (56.04)	525 (18.81)	251 (44.41)	1,275 (18.44)	1,020 (81.05)
Poorer	1,272 (26.76)	465 (48.57)	758 (27.15)	311 (52.66)	1,696 (24.52)	1,085 (82.98)
Poorest	1,355 (28.49)	527 (56.00)	875 (31.33)	369 (60.71)	1,906 (27.56)	1,112 (83.29)
Total	4,755	2,425	2,792	1,291	6,915	5,136

*Occupational numbers: frequencies do not add up to the total sum due to missing values.

**Figure 1.**
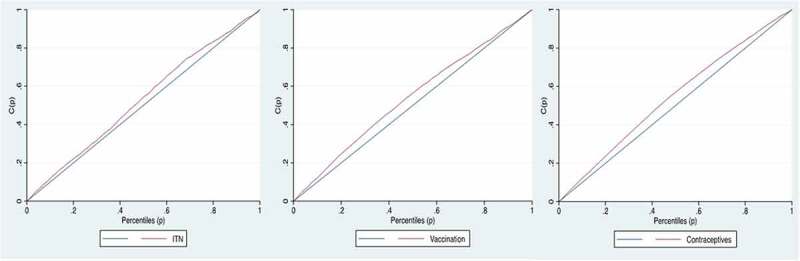
Concentration curves of the wealth inequality in lack of (a) ITN use; (b) vaccination coverage and, (c) modern contraceptive use.

In our sample, 43.2% of women belonged to the 15–24 years age group, and 74.73% were married. Almost two thirds of the participants lived in rural areas and were similarly distributed in the three regions of the country. One fourth of the women had no formal education, more than a half had primary education and less than a quarter had completed secondary school. The proportion of participants that reported not working was over half. Regarding the outcomes, more than half (51.01%) of women had at least one child under 5 years that did not sleep under an ITN, 46.25% had children that had not been fully immunised, and 74.28% of women were not using a modern contraceptive method.

he Cindex was −0.081 (95% CI: −0.11, −0.04; p-value: <0.01) for non-ITN use, −0.189 (95% CI: −0.23, −0.14; p-value: <0.01) for lack of full vaccination coverage and −0.284 (95% CI: −0.31, −0.24; p-value: <0.01) for non-contraceptive use, all indicating a pro-poor inequality illustrated by concentration curves above the line of equality (see Figure 1).

The summary results of the decomposition analysis, including the concentration indices, the coefficient estimates, elasticity, and their absolute and relative contributions to the total Cindex are presented in Table 2.

**Table 2.** Results of the decomposition of the concentration index(Cindex) of lack of ITN, vaccination coverage and modern contraceptive use

	Non ITN Use					No Full Vaccination					No Contraceptive Use				
				Contribution					Contribution					Contribution	
	Coef	Elast	Cindex	Abs	Rel	Coef	Elast	Cindex	Abs	Rel	Coef	Elast	Cindex	Abs	Rel
Age group (Ref 15–24)															
25–39	−0.009	−0.008	−0.072	0.001	−0.71	−0.045	−0.034	−0.019	0.001	−0.34	−0.079	−0.046	−0.053	0.002	−0.93
40–49	0.049	0.017	−0.012	0.000	0.24	−0.078	−0.025	−0.066	0.002	−0.88	0.017	0.004	−0.032	0.000	0.05
Marital Status (Ref Single)															
Married	−0.074	−0.097	−0.106	0.010	−12.64	−0.081	−0.098	0.003	0.000	0.17	−0.047	−0.044	−0.166	0.007	−2.74
Others	0.029	0.009	−0.144	−0.001	1.70	−0.072	−0.022	−0.157	0.003	−1.87	−0.034	−0.009	−0.123	0.001	−0.40
Subtotal demographic				0.010	−11.41				0.006	−2.92				0.010	−4.02
Residence (Ref Urban)															
Rural	0.058	0.074	−0.736	−0.055	67.45	−0.002	−0.003	−0.690	0.002	−1.09	−0.011	−0.009	−0.790	0.008	−2.91
Regions (Ref North)															
Centre	0.116	0.083	−0.239	−0.019	24.27	0.038	0.025	−0.225	−0.006	3.08	0.061	0.031	−0.226	−0.007	2.66
South	0.156	0.088	0.645	0.057	−69.69	−0.084	−0.044	0.610	−0.027	14.47	−0.077	−0.031	0.681	−0.021	8.01
Subtotal geographical			−0.330	−0.017	22.03				−0.031	16.46				−0.020	7.76
Education (Ref Secondary)															
Primary	0.077	0.078	−0.142	−0.011	13.67	−0.043	−0.040	−0.097	0.004	−2.09	0.089	0.065	−0.162	−0.010	3.96
No education	0.149	0.076	−0.402	−0.031	37.85	0.044	0.021	−0.402	−0.008	4.55	0.126	0.046	−0.438	−0.020	7.65
Occupational class (Ref Non manual)															
Farmers	0.042	0.018	−0.371	−0.007	8.00	0.061	0.024	−0.353	−0.008	4.49	0.031	0.009	−0.372	−0.004	1.33
Manual	−0.037	−0.004	0.176	−0.001	0.90	−0.003	0.000	0.190	0.000	0.03	−0.023	−0.002	0.197	0.000	0.14
Not working	0.035	0.038	0.069	0.003	−3.29	0.005	0.005	0.044	0.000	−0.11	0.064	0.049	0.039	0.002	−0.73
Wealth Quintile (Ref Richest)															
Richer	0.004	0.001	0.677	0.001	−1.04	−0.027	−0.008	0.701	−0.005	2.91	−0.004	−0.001	0.667	−0.001	0.24
Middle	0.063	0.023	0.367	0.008	−10.37	−0.006	−0.002	0.463	−0.001	0.54	0.047	0.012	0.277	0.003	−1.29
Poorer	0.047	0.023	−0.235	−0.005	6.57	0.049	0.022	−0.136	−0.003	1.63	0.099	0.035	−0.269	−0.009	3.54
Poorest	0.053	0.029	−1.020	−0.029	36.12	0.122	0.062	−1.083	−0.067	35.79	0.105	0.041	−1.000	−0.041	15.55
Subtotal socio economic				−0.072	88.41				−0.088	47.74				−0.080	30.39
Inequality Total				−0.08	100.0				−0.185	100.0				−0.263	100.0
Residual				−0.001	0.97				−0.072	38.72				−0.173	65.87
Inequality Explained				−0.079	99.03				−0.113	61.28				−0.090	34.13

Coeff: coefficient; Elast: elasticity; Cindex: concentration index; abs: absolute contribution, relative contribution.

**Figure 2.**
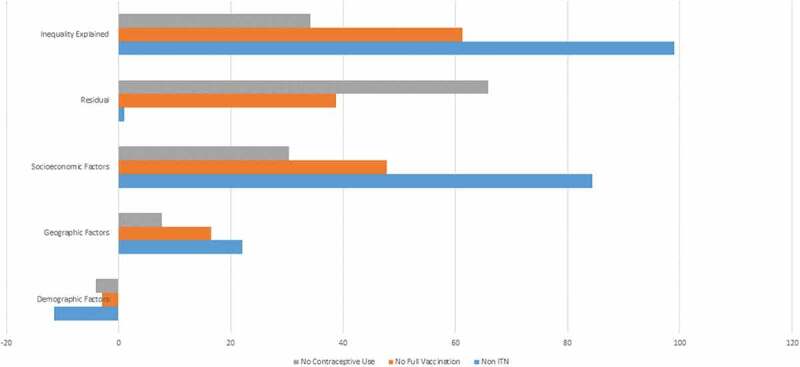
Contributions of demographic, geographical and socioeconomic factors to the wealth inequalities in the three reproductive and child health preventive measures.

The decomposition of the Cindex indicated that 99.03% of the wealth inequality in ITN use, 61.28% in vaccination and 34.13% in contraceptive use were explained by the sociodemographic, geographical, and socioeconomic variables included in the analysis (see Table 2 and Figure 2).

The analyses showed that 88.41% of the wealth gap in lack of ITN use were explained by socioeconomic factors, with education (37.85%) and wealth (36.12%) playing the largest role. Geographical factors came next in importance, explaining 22.03% of the inequality, while the sociodemographic factors (age and marital status) counteracted the effect of inequality (−11.41%).

Similarly, socioeconomic factors made most of the contribution in the case of lack of full vaccination (47.74%), mainly through the wealth variable itself (35.79%). Geographical factors played for this outcome a lesser role (16.46%), and the demographic variables had a small counteracting effect (−2.92%).

Finally, for the lack of contraceptive use, socioeconomic factors continued to be the main explanatory factors of the inequality (30.39%), but to a lesser degree than for the other two outcomes, with wealth (15.55%) and education (7.65%) contributing most to explaining the gap. Both geographical factors explained 8% of the gap, while the sociodemographic factors (age and marital status) slightly counteracted the effect of inequality (−4.5%).

## Discussion

To our knowledge, this is the first study decomposing wealth-related inequalities in health preventive care (ITN, vaccination, contraceptive methods) in Mozambique. Our study revealed a pro-poor inequality, implying that women from poor households were disadvantaged in the use of these three services compared to their richer counterparts. The inequality in the three health preventive care outcomes was, to different degrees, explained by the socioeconomic (wealth index, occupation, and education) and geographical (region and place of residence) determinants.

These results are in line with a broad literature from SSA, showing a higher risk among poor people of not using health care services, such as a lack of ITN, vaccinations and contraceptives in Mozambique [21], contraceptive use in Nigeria and Ghana [8], TB and HIV in South Africa [37], and antenatal care in Ethiopia [38], pointing out the existence of health inequalities disfavouring the less affluent population.

This study was able to explain 99.03% of the inequality in ITN, 61.28% in vaccination and only 34.13% in contraceptive use. Among all the variables, wealth and education were the predominant determinants of the overall wealth inequality in our three health preventive measures. Wealth has been reported to be the major contributor of the socioeconomic inequality in different maternal and child health outcomes in several countries of SSA such as in Nigeria [39], Malawi [40], Ghana [41] and Ethiopia [42]. Another study conducted in 32 SSA countries highlighted maternal educational level and household wealth as the main contributors to the wealth inequality in the mortality of children under five [6].

These results evidence that poor and low educated women have less access to these three free preventive measures. A combination of factors such as male-centric household decision-making, long distances to the nearest health facility, transportation costs and weak health service delivery have been discussed in the literature nationally [43,44]. In addition, the limited availability of maternal health services, qualified health professionals and medicines in many African countries is significantly undermining the health-care delivery to the most disadvantaged subgroups.

While a series of health reforms implemented over the last decades, such as increased investment in health infrastructure and workforce expansion and retention, might have improved access to health-care services, the contributors to the wealth gap found in this study call for efforts to be concentrated on increasing access to education and to income-generating programs, particularly among disadvantaged women.

Living in rural areas, and in the northern and central regions were also factors contributing to the wealth inequality. A possible explanation for the role of rurality could be that poor women living in rural areas often have to walk several kilometres to access health services and might not be able to afford transport costs; harvesting activities might also restrict their mobility in certain seasons [44]. Our findings are in line with a study from Ghana, where living in a rural area significantly contributed positively to the observed inequality in skilled birth attendants between 1998 and 2014 [45].

Previous studies in Mozambique have also reported geographic inequalities in resource allocations, with the privileged Southern region obtaining significantly more economic investment, including better social services such as health care and schools [46,47]. A better social and economic re-distribution of these resources within the country could contribute to reducing the observed wealth gap in health measures.

Finally, our study also revealed that sociodemographic factors (age and marital status) slightly counteracted the inequality in the outcomes, which can be explained by the more frequent use of the three preventive measures among married and middle-aged women in our sample.

## Methodological considerations

One of the strengths of the study was the use of the standardized IMASIDA questionnaire applied to a nationally representative sample. This approach makes our findings generalizable nationwide and comparable to studies from other countries using a similar survey instrument. In Africa, land possession is an important asset that should be taken into consideration when analysing wealth. However, variables used for the wealth index did not include land, which underestimates its role and importance for understanding African household wealth dynamics.

A negative value of the concentration index implies that a variable (here: the preventive health measure) is concentrated among the poor (pro-poor inequality), while the opposite is the case for a positive value (pro-rich inequality).

Since the independent and outcome variables were self-reported, recall bias could be operating. However, it was not possible to determine the extent of the bias. In addition, the question regarding modern contraceptive use at last intercourse did not capture a voluntary election for not using them.

Although our model explained most of wealth inequalities for ITN, the unexplained components for contraceptive use (65.87%) and for vaccination (38.72%) point to the need for further studies specifically designed to capture the contributory factors to the inequality in these outcomes. For instance, relevant variables such as community culture and women’s status, and the male dominant role in household decision-making might explain the wealth-health inequalities but were not included in the available dataset.

## Conclusion

This study provides evidence of considerable wealth-related inequalities in reproductive and child health in Mozambique, disfavouring less affluent populations. Findings suggest that the greater part of this inequality can be attributed to wealth itself, but also to education and residence in rural areas. We recommend that investment should be channeled into poor and non-educated rural communities to tackle these persistent inequities in health care use. Access to needed services is an essential step towards reducing wealth disparities in health and well-being between population sub-groups in the country.

## Paper context

Socioeconomic factors, particularly poverty, are associated with the use of health care services in Mozambique. Less is known about the determinants explaining socioeconomic differences. This paper presents wealth inequalities in insecticide treated bed-nets, child vaccination and modern contraceptive use disfavouring the poor, and reveals wealth, education, and rurality as key determinants of those inequalities. Results suggest the need to channel resources to the non-educated, poor rural communities to reduce wealth inequalities in preventive health care measures.

## References

[1] Emerson E. Relative child poverty, income inequality, wealth, and health. JAMA. 2009;301:425–9. 19176444 10.1001/jama.2009.8

[2] Wado YD, Sully EA, Mumah JN. Pregnancy and early motherhood among adolescents in five East African countries: a multi-level analysis of risk and protective factors. BMC Pregnancy Childbirth. 2019;19:59. 30727995 10.1186/s12884-019-2204-z PMC6366026

[3] Kravdal Ø. New evidence about effects of reproductive variables on child mortality in sub-Saharan Africa. Popul Stud. 2018;72:139–156. 10.1080/00324728.2018.1439180 29521576

[4] Moschovis PP, Wiens MO, Arlington L, et al. Individual, maternal and household risk factors for anaemia among young children in sub-Saharan Africa: a cross-sectional study. BMJ Open. 2018;8:e019654. 10.1136/bmjopen-2017-019654 PMC5961577 29764873

[5] Bradshaw CJ, Otto SP, Mehrabi Z, et al. Testing the socioeconomic and environmental determinants of better child-health outcomes in Africa: a cross-sectional study among nations. BMJ Open. 2019;9:e029968. 10.1136/bmjopen-2019-029968 PMC6773304 31570408

[6] Van Malderen C, Amouzou A, Barros AJ, et al. Socioeconomic factors contributing to under-five mortality in sub-Saharan Africa: a decomposition analysis. BMC Public Health. 2019;19:760. 31200681 10.1186/s12889-019-7111-8 PMC6570834

[7] Ogundele OJ, Pavlova M, Groot W. Inequalities in reproductive health care use in five West-African countries: a decomposition analysis of the wealth-based gaps. Int J Equity Health. 2020;19:44. 32220250 10.1186/s12939-020-01167-7 PMC7099835

[8] Ogundele OJ, Pavlova M, Groot W. Examining trends in inequality in the use of reproductive health care services in Ghana and Nigeria. BMC Pregnancy Childbirth. 2018 Dec 13;18:492. 30545328 10.1186/s12884-018-2102-9 PMC6293518

[9] McKinnon B, Harper S, Kaufman JS. Who benefits from removing user fees for facility-based delivery services? Evidence on socioeconomic differences from Ghana, Senegal and Sierra Leone. Soc Sci Med. 2015;135:117–123. 25965892 10.1016/j.socscimed.2015.05.003

[10] Ndwandwe D, Uthman OA, Adamu AA, et al. Decomposing the gap in missed opportunities for vaccination between poor and non-poor in sub-Saharan Africa: a multicountry analyses. Hum Vaccin Immunother. 2018;14:2358–2364. 29688133 10.1080/21645515.2018.1467685 PMC6284496

[11] Adeyanju O, Tubeuf S, Ensor T. Socio-economic inequalities in access to maternal and child healthcare in Nigeria: changes over time and decomposition analysis. Health Policy Plann. 2017;32:1111–1118. 10.1093/heapol/czx049 28520949

[12] Alkema L, Chou D, and Gemmill A, et al. Trends in maternal mortality: 1990 to 2013-estimates by WHO, UNICEF, UNFPA, The World Bank, and the United Nations population division. Geneva 27, Switzerland: The World Bank; 2014.

[13] World Health Organization. Trends in maternal mortality: 1990 to 2013: estimates by WHO, UNICEF, UNFPA, The World Bank and the United Nations population division: executive summary. Geneva 27, Switzerland: World Health Organization; 2014.

[14] DFID. Improving sexual, reproductive, maternal, newborn, child and adolescent health in Mozambique. Maputo: Mozambique DFID; 2017.

[15] Tsui AO, Brown W, Li Q. Contraceptive practice in sub-Saharan Africa. Popul Dev Rev. 2017;43:166. 29081552 10.1111/padr.12051 PMC5658050

[16] Ministerio da Saude. Relatorio da revisao do sector de saude Maputo. Mocambique Ministerio da Saude; 2014.

[17] Ministério da Saúde (MISAU) INdEI, and ICF. Survey of indicators on immunization, malaria and HIV/AIDS in Mozambique 2015: supplemental report incorporating antiretroviral biomarker results. Maputo (Mozambique); Rockville (MD): INS, INE, and ICF; 2015.

[18] Chavane LA, Gonçalves C. Inequalities in maternal and child health in Mozambique: a historical overview. UK: Institute of Development Studies; 2019. (IDS Working Paper).

[19] Da Silva T, Andrade X. Beyond inequalities: women in Mozambique. Harare (Zimbabwe): Southern African Research and Documentation Centre (SARDC); 2000.

[20] Llop-Girones A, Julia M, Chicumbe S, et al. Inequalities in the access to and quality of healthcare in Mozambique: evidence from the household budget survey. Int J Qual Health Care. 2019 Oct 31;31:577–582. 30388229 10.1093/intqhc/mzy218

[21] Daca C, Sebastian MS, Arnaldo C, et al. Socio-economic and demographic factors associated with reproductive and child health preventive care in Mozambique: a cross-sectional study. Int J Equity Health. 2020 Nov 9;19:200. 33168017 10.1186/s12939-020-01303-3 PMC7653841

[22] Macassa G, Burström B. Determinants of social inequalities in child mortality in Mozambique: what do we know? What could be done? Afr J Health Sci. 2005;12:118–121. 2011. 10.4314/ajhs.v13i1.30829 17348755

[23] Macassa G, Hallqvist J, Lynch JW. Inequalities in child mortality in sub-Saharan Africa: a social epidemiologic framework. Afr J Health Sci. 2011;18:14–26.

[24] Salvucci V. Determinants and trends of socioeconomic inequality in child malnutrition: the case of Mozambique, 1996–2011. J Int Dev. 2016;28:857–875.

[25] World Health Organization. A WHO report on inequities in maternal and child health in Mozambique. 2007.

[26] INE. Censo 2017 divulgação dos resultados preliminares. Maputo (Mozambique): INE; 2017.

[27] Stipp H. Extreme poverty rate in Mozambique 2016–2025. Statista; 2021 [cited 2021 Nov 25]. Available from: https://www.statista.com/statistics/1243825/extreme-poverty-rate-in-mozambique/

[28] Cunguara B. An exposition of development failures in Mozambique. Rev Afr Politics Econ. 2012;39:161–170.

[29] World Bank. Mozambique economic report a two speed economy. Maputo (Mozambique): World Bank; 2017.

[30] MISAU. Diploma Ministerial 127/2002, de 31 de Julho (Regulamento que define a caracterização técnica e enunciado das funções do Serviço Nacional de Saúde), BR n. 14, Suplemento. Maputo (Mozambique): Ministério da Saúde; 2002.

[31] World Health Organization. State of inequality reprodutive, maternal, newborn and child health. Geneva: WHO; 2015.

[32] Wagstaff A, Bilger M, Sajaia Z, et al. Health equity and financial protection: streamlined analysis with ADePT software. New York (NY): The World Bank; 2011.

[33] Nour TY, Farah AM, Ali OM, et al. Predictors of immunization coverage among 12–23 month old children in Ethiopia: systematic review and meta-analysis. BMC Public Health. 2020;20:1–19. 33243208 10.1186/s12889-020-09890-0 PMC7689978

[34] Croft TN, Marshall AMJ, and Allen CK, et al. Guide to DHS statistics DHS7. Rockville (MD): USA: ICF; 2018.

[35] Wagstaff A, van Doorslaer E. Overall versus socioeconomic health inequality: a measurement framework and two empirical illustrations. Health Econ. 2004 Mar;13:297–301. 14981654 10.1002/hec.822

[36] Wagstaff A, Doorslaer V, Watanabe N. On decomposing the causes of health sector inequalities with an application to malnutrition inequalities in Vietnam. New York (NY): The World Bank; 2001.

[37] Ataguba JE, Akazili J, McIntyre D. Socioeconomic-related health inequality in South Africa: evidence from general household surveys. Int J Equity Health. 2011;10:1–10. 22074349 10.1186/1475-9276-10-48 PMC3229518

[38] Bobo FT, Yesuf EA, Woldie M. Inequities in utilization of reproductive and maternal health services in Ethiopia. Int J Equity Health. 2017 June 19;16:105. 28629358 10.1186/s12939-017-0602-2 PMC5477250

[39] Nwosu CO, Ataguba JE. Socioeconomic inequalities in maternal health service utilisation: a case of antenatal care in Nigeria using a decomposition approach. BMC Public Health. 2019;19:1493. 31703734 10.1186/s12889-019-7840-8 PMC6842188

[40] Chirwa GC, Mazalale J, Likupe G, et al. An evolution of socioeconomic related inequality in teenage pregnancy and childbearing in Malawi. PLoS One. 2019;14:e0225374. 31747437 10.1371/journal.pone.0225374 PMC6867649

[41] Novignon J, Ofori B, Tabiri KG, et al. Socioeconomic inequalities in maternal health care utilization in Ghana. Int J Equity Health. 2019;18:141. 31488160 10.1186/s12939-019-1043-x PMC6729067

[42] Shifti DM, Chojenta C, Holliday EG, et al. Socioeconomic inequality in short birth interval in Ethiopia: a decomposition analysis. BMC Public Health. 2020;20:1504. 33023567 10.1186/s12889-020-09537-0 PMC7542382

[43] Dos Anjos Luis A, Cabral P. Geographic accessibility to primary healthcare centers in Mozambique. Int J Equity Health. 2016 Oct 18;15:173. 27756374 10.1186/s12939-016-0455-0 PMC5070361

[44] Jani JV, De Schacht C, Jani IV, et al. Risk factors for incomplete vaccination and missed opportunity for immunization in rural Mozambique. BMC Public Health. 2008;8:161. 18485194 10.1186/1471-2458-8-161 PMC2405792

[45] Abekah-Nkrumah G. Spatial variation in the use of reproductive health services over time: a decomposition analysis. BMC Pregnancy Childbirth. 2018;18:63. 29510675 10.1186/s12884-018-1695-3 PMC5838884

[46] Bujones AK. Mozambique in transition and the future role of the UN. Center on International Cooperation; 2013.

[47] O’Laughlin B. Questions of health and inequality in Mozambique. Maputo (Mozambique); 2010. (Instituto de Estudos Sociais e Económicos Cadernos IESE No. 4).

